# Social Media and Online Digital Technology Use Among Muslim Young People and Parents: Qualitative Focus Group Study

**DOI:** 10.2196/36858

**Published:** 2022-05-10

**Authors:** Caitlin H Douglass, Aidan Borthwick, Megan S C Lim, Bircan Erbas, Senem Eren, Peter Higgs

**Affiliations:** 1 Burnet Institute Melbourne Australia; 2 Melbourne School of Population and Global Health University of Melbourne Melbourne Australia; 3 La Trobe School of Psychology and Public Health La Trobe University Bundoora Australia; 4 Monash School of Public Health and Preventive Medicine Monash University Melbourne Australia; 5 Green Crescent Australia Melbourne Australia; 6 School of Humanities and Social Sciences Ibn Haldun University Istanbul Turkey

**Keywords:** Muslim, social media, young adult, qualitative research, social connection, parenting, pediatrics, digital health, youth, adolescent, parent, digital technology, user experience, mental health, psychological effect, diverse population, COVID-19

## Abstract

**Background:**

Digital technology and social media use are common among young people in Australia and worldwide. Research suggests that young people have both positive and negative experiences online, but we know little about the experiences of Muslim communities.

**Objective:**

This study aims to explore the positive and negative experiences of digital technology and social media use among young people and parents from Muslim backgrounds in Melbourne, Victoria, Australia.

**Methods:**

This study involved a partnership between researchers and a not-for-profit organization that work with culturally and linguistically diverse communities. We adopted a participatory and qualitative approach and designed the research in consultation with young people from Muslim backgrounds. Data were collected through in-person and online focus groups with 33 young people aged 16-22 years and 15 parents aged 40-57 years. Data were thematically analyzed.

**Results:**

We generated 3 themes: (1) maintaining local and global connections, (2) a paradoxical space: identity, belonging and discrimination, and (3) the digital divide between young Muslims and parents. Results highlighted that social media was an important extension of social and cultural connections, particularly during COVID-19, when people were unable to connect through school or places of worship. Young participants perceived social media as a space where they could establish their identity and feel a sense of belonging. However, participants were also at risk of being exposed to discrimination and unrealistic standards of beauty and success. Although parents and young people shared some similar concerns, there was a large digital divide in online experiences. Both groups implemented strategies to reduce social media use, with young people believing that having short technology-free breaks during prayer and quality family time was beneficial for their mental well-being.

**Conclusions:**

Programs that address technology-related harms must acknowledge the benefits of social media for young Muslims across identity, belonging, representation, and social connection. Further research is required to understand how parents and young people can create environments that foster technology-free breaks to support mental well-being.

## Introduction

Digital technology and social media have changed how people interact and build social connections [1]. Social media sites, such as Instagram, Facebook, SnapChat, YouTube, and TikTok, have become increasingly popular and enable young people to relax, seek information, and construct and maintain their social networks [2,3]. Since the COVID-19 pandemic, social media use among young people has increased, particularly during lockdown periods, when socializing outside of the home was heavily restricted [4,5]. Although routine social media use has been associated with higher levels of social well-being and positive mental health [6], emotional connection to social media, excessive use, and passive browsing have been associated with decreased social well-being and poorer mental health outcomes [7-9]. However, these findings are heterogenous, and few studies have adopted longitudinal designs; thus, it is unclear whether social media has negative effects on well-being [10,11]. Although these quantitative data are important, they do not provide insight into the experiences that young people have online.

Qualitative research has explored young people’s use of digital technology and social media to access news, develop relationships, and form their identity [12-15]. Parents have also documented their concerns about their children spending time online, fearing they will be exposed to online bullying, isolation from the outside world, and negative mental health outcomes [16,17]. However, few studies have focused on the online experiences of young people and parents from cultural and ethnic minority groups. Minority populations have experienced racism and discrimination online, including Islamophobic hate speech, targeted bullying, and threats of offline violence for people from Muslim backgrounds [18-20]. Importantly, young people from cultural and ethnic minority groups also use social media to access social support and connect with family who live overseas [21,22]. Young people from Muslim backgrounds have also described how online environments promote agency, social connection, inclusion, and expression of identity and religion [23-27]. Few qualitative studies have recruited both young people and parents from cultural and ethnic minority groups; thus, further research is required to better understand their experiences on social media, particularly during the COVID-19 pandemic.

In Australia, the 2016 census reported that 3% of the population identified as Muslim [28]. Approximately 37% of this group were born in Australia and 63% were born overseas across countries in North Africa, the Middle East, and South and Central Asia [29]. In 2019, Green Crescent Australia, a not-for-profit organization that works closely with Muslim community members in Melbourne, Victoria, Australia, approached researchers and indicated that community members had expressed concern about technology dependence among young people. These concerns were predominantly from parents and older community members; however, it was unclear whether concerns reflected the experiences of young people from Muslim backgrounds. To develop recommendations, programs, and policies that are relevant for families, it is essential to listen to both young people and parents from Muslim backgrounds and promote their voices and experiences [30,31]. In this exploratory study, we aim to understand the positive and negative experiences of digital technology and social media use from the perspective of young people and parents from Muslim backgrounds.

## Methods

### Partnership

This exploratory study involved a partnership between Green Crescent Australia, the Burnet Institute, and La Trobe University. Green Crescent Australia is a not-for-profit organization that provides culturally intelligent education and awareness programs for substance and behavioral dependencies in culturally and linguistically diverse communities. The Burnet Institute is a not-for-profit medical research organization with experience in exploring social media use of young people. La Trobe University has research programs exploring diverse public health issues. Each partner brought unique skillsets to the study, including community connections, cultural knowledge, and youth-friendly research skills.

### Study Design and Development

Green Crescent Australia approached the researchers to develop a study exploring technology dependence among young Muslims based on concerns from parents that arose during regular community meetings. We developed the study design and research questions through face-to-face and phone meetings between the study partners. Based on the researchers’ experience of conducting studies with young people and reading background literature, we suggested exploring both negative and positive experiences of social and digital media from the perspective of young people and parents from Muslim backgrounds. Overall, we adopted a participatory and qualitative approach that was appropriate for the study aim of understanding participant experiences with social media. The research team held 2 sessions with Green Crescent Australia representatives (4 adults and 10 young people) to present ideas, pilot-test activities, and obtain feedback on the study design. Overall, the representatives believed their peers would be comfortable discussing digital technology and social media in small groups with people of similar age and gender, thus leading to the use of focus groups. Focus groups were deemed appropriate because interactions between participants can provide further insight into their experiences with social media [32]. Both adult and youth volunteers suggested to make the focus groups culturally appropriate by including halal food options, dressing appropriately, building in time for prayer breaks, and holding the focus groups at venues familiar to participants (eg, mosques, schools).

### Ethics Approval

The La Trobe University Human Research Ethics Committee approved the study (#HED 19458).

### Participants and Recruitment

Young people were eligible to participate in the study if they were aged between 16 and 22 years and identified as Muslim. This age range was selected to account for the researchers’ past experiences where wide age gaps between participants limited the ability of younger participants to contribute to the discussion. Parents were eligible if they identified as Muslim and had children aged 13-22 years; this age range was chosen as we anticipated some shared concerns from parents of teenagers and young adults. Parents did not have to be related to a young person in the study to be eligible. Because the Green Crescent Australia representatives were part of the Muslim community, they assisted in participant recruitment. We used purposive sampling to ensure that we recruited participants who represented different genders, age groups, and ethnicities and were able to provide relevant information about the topic. Determining our sample size was a pragmatic exercise [33]; we aimed to recruit between 40 and 50 participants based on the study time frame and budget and to collect data from a diverse sample. Green Crescent Australia representatives contacted Muslim youth organizations, Islamic schools, and their social networks to describe the study’s purpose. Those who were interested were sent a copy of the participant information sheet and consent form prior to the focus group.

### Data Collection

We held 7 focus groups of 90 minutes each between November 2019 and September 2020, including 4 with only young people, 2 with only parents, and 1 with both young people and parents. The first 4 focus groups were in person at local mosques and community-based venues in the cities of Melbourne and Hume. Most participants who attended the focus groups knew one another beforehand. During data collection, Melbourne went into 112 days of COVID-19 lockdown; thus, we submitted an ethics amendment so that the 3 remaining focus groups could occur online through the videoconferencing platform Zoom [34]. Researchers facilitated the focus groups in English, with support from a Green Crescent Australia representative who could speak Arabic. A female researcher facilitated the female focus groups, and 2 male researchers facilitated the male focus groups. One exception was 1 male participant who attended a predominantly female focus group with a relative and friends. Prior to the focus group starting, the researchers verbally explained the study and provided an opportunity for questions. Participants were invited to provide written informed consent for in-person focus groups and verbal consent for online focus groups.

The focus groups began with an icebreaker activity, where participants and facilitators shared the meaning of their name. We asked young people and parents to list how they or their children used digital technology throughout the day (eg, what devices, programs, websites, social media sites, and apps). This activity generated discussion between participants and was followed up with the prompt “What are the positive and negative impacts of digital technology or social media on your or your children’s lives?” After the first 2 focus groups, the researcher adapted the focus group guide to include additional prompts about strategies to reduce technology use, as participants brought this up of their own accord in the first 2 focus groups. When focus groups were in person, participants recorded their responses on sticky notes and added them on to a whiteboard compared to Zoom focus groups where we used an interactive online whiteboard. Written responses were used to generate discussion, and participants could elaborate on their answers. Upon completion, participants received AU $40 (US $28.44) in cash or were sent an AU $40 (US $28.44) voucher if they attended online focus groups.

### Data Analysis

Focus groups were audio-recorded and transcribed verbatim. Personal identifying information was removed from the transcripts. We coded and analyzed the data thematically using a descriptive approach [35]. This process involved reading the transcripts multiple times and reviewing the written and electronic material produced by the participants. Transcripts were coded in NVivo (QSR International) [36]. We adopted an inductive approach to coding and generated ideas from the raw data rather than using a codebook. Coding focused on understanding the meanings and experiences that participants ascribed to digital technology and social media. We also noted where there were key differences between participant groups (eg, gender, parents/young people, before or during COVID-19). In total, 6 preliminary themes were summarized and presented verbally to coauthors for feedback. Discussions indicated that although preliminary themes were relevant to the research question, there was some overlap between ideas. Consequently, the first author placed all codes into a mind map, reviewed the transcripts, and rearranged the data into 3 key themes. Coauthors provided additional feedback on the content included in each theme.

### Researcher Positionality

In this study, the research team consisted of 2 Muslim and 4 non-Muslim researchers. The Green Crescent Australia representatives involved in study development, participant recruitment, and data collection and interpretation also identified as Muslim. Data analysis was led by a non-Muslim researcher; thus, obtaining feedback from the broader research team was essential to ensure we considered religious and cultural factors that may have been missed. In addition, 2 researchers were under 30 years old, 2 were parents, and all used social media; thus, each individual brought a unique perspective to data interpretation.

## Results

### Participants

Overall, we conducted 7 focus groups with 48 participants, including 33 (69%) young people and 15 (31%) parents. Most of the young participants were female (n=25, 76%) and born in Australia (n=28, 85%), whereas most of the parent participants were male (n=10, 67%) and born overseas (n=9, 60%). Participants identified with a range of ethnicities, including Turkish, Australian, Somali, Albanian, Ethiopian, Bosnian, Lebanese, Palestinian, Pakistani, African, Yemeni, South Asian, Eritrean, Egyptian, and Jordanian. From these focus groups, we generated 3 key themes to showcase the positive and negative impacts of digital technology and social media from the perspective of young Muslims and parents: maintaining global and local connections, a paradoxical space: identity, belonging and discrimination, and the digital divide between young Muslims and parents.

### Maintaining Global and Local Connections

Theme 1 highlighted how participants perceived online technology as an important environment for social connection both before and during the COVID-19 pandemic. Participants described how social media acted as a space for developing and maintaining social connections in Australia and across national boundaries. Young people described how their online interactions were typically extensions of their friendships from real life; they could spend most of the day with their peers at school or university and then continue interacting online to strengthen their relationships. For both young Muslims and parents, social media and group messaging platforms were seen as practical tools that enabled them to overcome distance and connect with their friends and family who lived overseas.

Now we can instantly message someone. You used to have to send a letter to someone on the other side of the world. You save a lot of time, you know?

Young male, online focus group

Having access to a phone and being constantly contactable by friends and family increased social security for young male and female participants. Although this was not apparent in the parent focus groups, researchers noted that almost all participants checked their phones at least once during the in-person focus groups. Young participants also described being frequently called and messaged by their parents and other family members to ensure they knew their whereabouts. Young participants rarely left home without their phones, expressing an anxiety that they may miss key events or updates in their friendship groups, demonstrating that access to technology is tied to social connection. When asked how they felt when they did not have their phone, a common response was:

Definitely separated, like, left out, you know? Disconnected in a sense.

Young male, online focus group

Among female participants, always carrying a phone and being able to contact people they trusted represented an increased sense of safety. For example, some female participants felt that carrying their phone was responsible and a mechanism for protecting themselves. This topic did not come up during male focus group discussions.

My phone…if I press the trigger lots of times, it sends an SOS. So, it will send my location to specific people that I want to send it through. So, I have it on for my cousins and my close friends.

Young female, in-person focus group 1

Online focus groups held during the Melbourne lockdown period drew attention to the isolation that people experienced. Although participants used social media to connect with friends and family who were outside of their households, both young people and parents felt disconnected during the lockdown that coincided with Eid, a significant religious festival where Muslim community members typically gather for prayer and share food, gifts, and donations. One parent described Islam as a “social religion”; therefore, being unable to connect in person at places of worship was highly difficult. Young people also expressed missing their routine, in-person social connections that would typically occur through school, work, or university.

For the past 6 months have not being able to go to the mosque. We can’t go. If you go to the mosque, you see people, you say hello, you find out who is suffering, who has problems, you attempt to resolve them. We help each other out as a community. Technology has not reached that point yet.

Father, online focus group 7

Overall, prior to the pandemic, it was essential for young people to physically have their phones to feel a sense of social security and physical safety, particularly among young females. After COVID-19 caused long periods of lockdown and physical distancing, having access to technology and social media became essential for both young people and parents to socially connect with the outside world; however, some parents felt that technology was insufficient to generate the sense of community they felt when they attended mosque.

### A Paradoxical Space: Identity, Belonging, and Discrimination

Theme 2 captured how online environments were paradoxical spaces where young Muslims could establish their identity and belonging while also being exposed to discrimination. Young female participants described how online spaces, such as Instagram and TikTok, allowed them to curate communities where they felt comfortable and safe. Young male participants did not discuss comfort and safety; however, they appreciated being able to express their opinions and follow people they admired online. Parents, particularly mothers, also recognized that using social media was considered “normal” for young people and a tool for “fitting in.” When female participants followed accounts that they considered positive, they felt a sense of creativity, inspiration and belonging.

I feel like it curates, like you have your own little community. Like, um, with my social media, most of my followers, they're all, like, Muslim, so I feel like I know all of them and I feel comfortable with them, so it's having that support from them.

Young female, online focus group 6

I definitely do try and follow more pages of people that look like me and have the same interests as me. I follow pages that are, like, pro-African, pro-Black, like, pro-Islam, [because] that's [the] kind of the thing that we don't get to see anywhere else.

Young female, focus group 4

Young people also felt that social media enabled them to see their culture, religion, and minority groups represented. Multiple participants felt this representation and diversity was lacking in mainstream media, which was perceived as biased and one-dimensional. Although mainstream media was seen as an exclusive environment that promoted the agendas of powerful and privileged groups, social media was seen as more inclusive for minority groups. Female participants in the online focus group were particularly passionate about this topic, which coincided with global news about the murder of George Floyd and widespread advocacy on social media.

Often with regular news, you’ll only get a single perspective. With the internet and talking to other people, you get a different understanding or a different point of view rather than what has just been given to you.

Young male, online focus group 5

We were stuck in lockdown during Eid. On social media, you can see, like, everyone…like posting about it. Even, like, when the Black Lives Matter everything happening, you see videos and photos from, like, people that went to protests, people advocating for Black Lives Matter, and as a Black person, you know you feel like you've got support, you've got representation.

Young female, online focus group 6

Despite positive experiences, both young people and parents recognized that digital technology has the potential to perpetuate discrimination, exclusion, and unrealistic standards around beauty and success. Parent participants had concerns about online bullying among young people, with mothers expressing fears about the nonconsensual sharing of sexually explicit images. A small number of young female participants echoed these concerns; however, most of the discussion centered on receiving negative comments on social media profiles. Other young people had seen more “passive” acts of discrimination, such as friends sharing, liking, or commenting on racist or Islamophobic posts or videos. Young participants believed the anonymity of the internet allows people to be discriminatory without consequences.

You can stay somewhat anonymous. With certain individuals, they go around spewing hate…They can speak in the world how they want, and they can get away with certain things. Things that they would not get away with in real life.

Young male, online focus group 5

Although social media could be a positive space, most young people compared their lives to the ideals portrayed online by celebrities and influencers. Female participants were frequently exposed to unrealistic beauty standards, which created self-doubt about their own appearance. Young female participants also expressed concern for future generations of young Muslim women who were exposed to social media earlier in life; they recognized that although they could curate their own online spaces, there was still limited beauty coverage inclusive of Muslim women and hijabs.

Little girls that are growing up now, they’re having these, like, expectations to dress in a certain way. Even with social media sometimes, there’s not a lot of coverage on females that wear scarves, and if a young child sees that, they just don’t feel like they have a place in society.

Young female, online focus group 6

I think it’s creating a lot of anxiety, too. My daughter recently went off TikTok because she said, “Everyone on there is so beautiful. They make me feel ugly.” Well, I said, you know, that’s probably a good time to get off it.

Father, in-person focus group 3

Although parents believed these online pressures were worse for young females, the young male participants also described seeing unrealistic standards of success that made them question their own goals and achievements. Although young male participants recognized that social media showed only the best parts of people’s lives, they still questioned their self-worth.

I think going off that also the fact that people hold certain ideal standards. For social media, for example, someone will only post the really great moments or those really perfect pictures. And they’re setting a standard that in reality is not achievable.

Young male, online focus group 5

Seeing people that are successful and all that on social media all the time, it kind of affects you because you think that you need to be there right now. You sit down and compare your life to other people's lives…they don't show their real lives…they're just showing the highlights, the best parts.

Young male, in-person focus group 4

These experiences highlight how digital technology can boost the identity and belonging of young Muslims while still being a risk environment for discrimination, self-doubt, and negative self-esteem. Importantly, being able to curate personal online spaces increased the representation of minority groups, which was perceived as lacking on other mainstream channels.

### The Digital Divide Between Young Muslims and Parents

Theme 3 outlined the distinctions between how young Muslims and parents defined legitimate and productive forms of technology use and appropriate strategies for reducing time spent online. Importantly, young participants focused on social media sites being mostly positive environments where negative events could occur. In contrast, parents focused on social media sites as negative and potentially dangerous spaces where positive connection was possible. Young people and parents recognized that their generations had been introduced to technology at different life stages, thus creating a major divide in their experiences. Parents perceived that spending time online robbed young people of the opportunity to exist in the “real world” that they had grown up in when they were children/adolescents. They discussed how young people were unaware of their prolonged use and could not see the negative impact it was having on their lives.

When we were young, we were always outside. Every day. We come home from school, always outside. Now, you look on the street and there is no one outside. My kids, I’ve got 2 younger boys, they’re always on YouTube watching other people play. I’m, like, go and do it yourself!

Father, in-person focus group 3

In these teen years, I feel that they're missing out on, like, actually life, like doing things, seeing things, and they have no interests, like, my kids, like, even holiday destinations…it's like, “Oh, but don't you wanna see this and this,” “'Nup, like, just Google it,” you know.

Mother, in-person focus group 2

In contrast, young people were cognizant of the large amounts of time that they spent online and the potentially negative outcomes. They perceived that increased screen time was a consequence of technology being embedded in their social, school, and work lives rather than a conscious choice. When technology was used for a clear purpose, such as learning, consuming news, exploring interests, expressing oneself, working, relaxing, or communicating, young people felt that this use was legitimate and acceptable. Although parents believed that online learning, exploring interests, and consuming news were productive forms of technology use for young people, they could not personally relate to social media being a place for identity and belonging.

When I was in high school, I was really depressed, and I had Tumblr. And for me, that was my way of, like, you share posts and quotes. And I remember my parents were, like, to me, “You can’t use Tumblr anymore.” And, I was so upset, I was, like, you don’t understand how I express myself.

Young female, in-person focus group 1

For both young people and parents, certain activities were associated with time wasting and productivity guilt. For young people, these activities included scrolling through apps, such as Instagram, without realizing how much time was passing; watching short videos on TikTok and YouTube for hours; constantly checking social media for no specific reason; and using their phones when they needed to concentrate on a different task. A minority of parents also described how they personally used social media in these nonproductive ways; however, this was perceived as far more common among their children.

I was getting really agitated when I couldn’t check it. I reckon everyone here does it. I’d go into Instagram…no updates. I’d go into Snapchat…no updates, and then by the time I’ve gone to Snapchat, I’ll be back on Instagram. And I’ll be, like, Oh, I just got out of it. So, I was going constantly back and forth.

Young female, in-person focus group 1

There's so many different platforms to keep them entertained, and then having said that, I know myself, I'm on Insta so much, the time I waste on that I could be doing a lot!

Mother, focus group 2

Both young people and parents agreed that reducing technology use was extremely difficult. Parents felt a sense of responsibility to protect their children from online harms and spending excessive amounts of time online. Often, their attempts to reduce technology use were punitive, for example, confiscating phones as punishment or turning off the Wi-Fi to enforce family time. Some parents felt compelled to monitor what their children were doing online by checking their personal profiles to see who they followed and what they shared. Some parents adopted a more hands-off approach, where they allowed their children to have freedom online but ensured they had regular conversations about what they were doing and the potential risks. Despite these attempts, parents recognized that their efforts were hindered because their children had greater digital literacy and could easily hide their history. At the end of the focus groups, parents commented how they had learned about new social media platforms from the other parents who attended the session and reflected on how they generally relied on their children to educate them about digital technology.

You keep tabs on them so much more like you've gotta be involved in their life and what's actually going on.

Mother, in-person focus group 2

I know, but, you know that, they're so, they'll only let you see what they want you to see.

Mother, in-person focus group 2

But, you know, my kids are on a lot of that social media stuff, and it makes me crazy. Every 2 seconds, you know? I’ll be sitting there talking to them, and there will be a beep, and they’ll grab their phone. Give me that bloody phone! If you take it off them, they become normal human beings.

Father, in-person focus group 3

For young people being told to “get off their phones” was unrealistic because their school, work, and social lives depended on their online presence. Although some young people had tried to cut back their screen time by turning off notifications, deleting apps, and setting time limits, these efforts were largely unsuccessful. However, there were certain times when young people were motivated to have short technology breaks, generally when they had a goal or activity that required them to focus or be present. For example, young people described having technology breaks when they were praying, spending quality time with close friends, or having meals with their families. During this technology-free time, young people would generally create physical distance from their phone so they did not feel obliged to check it. For young people, it was important that they were doing something enjoyable in a safe space when they did not have their phones, otherwise the separation led to anxiety.

If I’m praying…I realize that if my phone is in the room while I’m praying, as soon as I hear it ringing, I want to look at my phone. I make the effort to take time while I’m praying…whereas if it’s not there, I’m, like, I’ll stay an extra few minutes, do extra, take some more time.

Young female, in-person focus group 1

Although both young Muslim and parent participants reported using digital technology and social media, their perceptions of productive use differed. Participants recognized the difficulties of reducing screen time; however, capitalizing on the desire for technology-free breaks during prayer and family time warrants further investigation.

## Discussion

### Principal Findings

Our study highlights how digital technology and social media are ingrained into the routines and social worlds of young people and parents from Muslim backgrounds. Our results demonstrate that parents and young participants share some similar concerns about digital technology and social media; however, they have different online experiences, particularly around identity and belonging. Young people and parents considered digital technology and social media use as acceptable when they were used for a specific purpose. However, activities considered mindless, such as scrolling and constantly checking notifications, were associated with productivity guilt. Not surprisingly, young people felt their social media use increased during the COVID-19 lockdown period and valued having technology-free breaks while praying or spending quality time with family.

Participants acknowledged that technology has a myriad of benefits, including connecting with friends and family, facilitating online work and study, and providing spaces for identity and belonging. Social media allows young Muslims to explore their interests, express themselves, and create virtual social worlds where they feel safe and included [25]. Non-Muslim young people and parents have reported similar benefits of connection, participating in work and study and identity formation [37,38]. Our participants also described social media as more diverse than mainstream media; however, they hoped to see more positive representation of Muslim communities in the future. On a practical level, young people from Muslim backgrounds should be supported to participate in online environments where they feel connected, represented, and comfortable to explore their identity.

Importantly, our study also found that young people and parents have experienced and observed harms from digital technology and social media, including exposure to discrimination. Islamophobic hate speech on social media is a major concern and creates fear and exclusion among Muslim communities [20]. Social media platforms have a responsibility to identify and remove hate speech from their platforms; however, perpetrators rarely face consequences [39]. Our study showed that young Muslims are exposed to unrealistic standards on social media and often compare themselves to others, contributing to self-doubt and negative self-esteem. Building young people’s social media literacy to analyze and evaluate online content may protect against some of these harms; however, further research is needed [40].

Our study suggests there is a large divide in parents’ and young people’s online experiences. Parents believe that their children’s preference for technology-based leisure is inferior to the leisure activities they valued during their childhood and adolescence. These results are similar to studies with non-Muslim young people and parents that have reported that intergenerational differences in technology use can create family tension [1,37]. Some parents attempted to monitor their children’s use; however, their limited digital literacy made it difficult. Similarly, an online survey reported over a quarter of parents felt they lacked the computer skills to protect their children from technology-based harms [2]. Parents may benefit from digital literacy training so that they can support their children to participate in online environments while also mitigating potential harms [41]. Although most participants had implemented various strategies to reduce young people’s screen time, efforts had largely been ineffective. This finding reflects the broader literature that suggests that reducing recreational screen time among adolescents is difficult; results from a cluster-randomized controlled trial indicated that a 6-month intervention with reminder messages and education targeting both young people and parents had no significant impact on screen time [42]. Although reducing screen time was difficult, our participants valued having short technology breaks when they had a goal or activity where they wanted to feel present. Key activities that facilitated these breaks included prayer times and activities shared with family and friends where they felt safe and positive. In the Muslim community, prayer occurs 5 times per day and is considered a pillar of Islam; thus, integrating technology breaks around times of worship may be feasible. Further research is needed to determine whether reducing time spent on social media has a positive effect on mental health and well-being [43,44].

Since COVID-19, participants reported an increase in digital technology and social media use. This finding is similar to results from a survey with mostly non-Muslim young people, with 74% reporting that their social media use had increased during the pandemic and that it was the main way they stayed connected [45]. In our study, participants reflected that digital technology became the only place to work, study, and socialize during the lockdown. This shared experience meant that parents became more understanding of the time that young people spent online. Although young people acknowledged that social media enabled them to stay in touch with their friends, they missed human interaction. Parents also missed their routine social connections, as they were no longer able to attend the mosque during the lockdown. Qualitative research in the United Kingdom highlighted that although mosque closures were an important public health measure to reduce the spread of COVID-19, many Muslim community members lost their usual channels of communication [46]. Social media is an important extension of social connection; however, this medium does not replace the need for face-to-face interactions that occur in structured environments, such as school and places of worship. Further research on how faith groups can be supported to maintain a sense of community during COVID-19 lockdowns is warranted.

### Strengths and Limitations

This study had some limitations. Participants were recruited through the networks of the researchers and Green Crescent Australia and were often connected with mosques, Islamic schools, or youth groups. Most of the participants knew each other; thus, it is unlikely that we reached young people and parents who were socially isolated and those who did not attend the mosque. Most of the young participants were born in Australia and identified as female. In contrast, most of the parent participants were male; this likely occurred because we conducted a focus group with fathers on Zoom during the COVID-19 lockdown in Melbourne, which may have been more convenient to attend than the mothers’ focus group, which occurred in person prepandemic.

Our ability to adapt the mode of data collection was a strength of our research and enabled us to explore social media use among young Muslims and parents in a COVID-19-safe way. Although we anticipated it would be difficult to run online focus groups after participants had likely spent extended periods online, participants appeared to enjoy discussing and reflecting on their experiences. Additionally, a community organization drove this research, enabling us to establish strong links with the Muslim community, incorporate feedback, and increase the study’s cultural appropriateness by holding focus groups at local mosques, providing halal food, having prayer breaks, and being mindful of the gender of participants and facilitators. We were also able to communicate the findings with Green Crescent Australia to share with their community.

### Conclusion

This study provided insight into digital technology and social media use from the perspective of young people and parents from Muslim backgrounds. Programs that aim to address technology-related harms must acknowledge the benefits of digital technology and social media for young people for their identity, belonging, representation, and social connection, particularly during the COVID-19 pandemic. Young people and parents should be supported in developing digital literacy skills to enable participation in online environments, while mitigating potential harms. Further research is required to understand how parents and young people can create environments that foster technology-free breaks and the effect on mental well-being.
